# Focused Critical Care Billing Education and Feedback as Part of the Emergency Medicine Residency Curriculum Is Effective

**DOI:** 10.7759/cureus.77874

**Published:** 2025-01-23

**Authors:** Nick Schoenmann, Stanton Royer, William S Guyton, Hongyan Xu, Lifang Zhang, Maggie M Needham, Ann Marie Kuchinski, John R Barrett

**Affiliations:** 1 Department of Emergency Medicine, Medical College of Georgia, Augusta University, Augusta, USA; 2 Emergency Department, Huntsville Hospital, Huntsville, USA; 3 Department of Biostatistics, Data Science, and Epidemiology, Augusta University, Augusta, USA; 4 Department of Population Health Sciences, Geisinger Medical Center, Danville, USA; 5 Department of History, Anthropology, and Philosophy, Augusta University, Augusta, USA

**Keywords:** billing, coding, critical care, documentation, education intervention, emergency medicine, medical education, residency curriculum

## Abstract

Background: Emergency medicine physician compensation models often have a productivity component based on the relative value unit. The primary goal of this study was to determine if implementing critical care billing education through both lectures and individual resident feedback as a formal part of the emergency medicine residency curriculum improved critical care billing among residents.

Methods: This was a prospective, pre and post, educational intervention, cohort study of emergency medicine resident critical care billing in an academic emergency department. The study period was from July 2019 through June 2021. Critical care billing data obtained for the academic year of July 2019 through June 2020 established a baseline of resident critical care billing practices. Beginning in April 2020, monthly emails were sent out to the residents listing their critical care billing percentage for each month. The intervention began in July 2020 with a dedicated critical care billing lecture, which was repeated in October 2020. Average critical care billing percentages were discussed with residents during semi-annual evaluations in December 2020 and May 2021. Critical care billing data obtained for the academic year from July 2020 through June 2021 assessed these interventions.

Results: Of 44,438 patients seen by residents in the baseline year of the study from July 2019 through June 2020, an average of 5.16% or 2,456 patients were accurately billed for critical care services by residents. The following academic year, after critical care billing interventions were added to the residency curriculum, the accurately billed critical care rate increased to 10.66%, to 4,304 of the 39,396 patients seen by residents. Patients for whom critical care billing was attempted by residents but criteria were not met decreased slightly over the study period, from 666 or 1.43% patients in the baseline year to 529 or 1.31% patients the following year in a non-statistically significant manner.

Conclusion: Focused critical care billing educational interventions as part of the emergency medicine residency curriculum improved the accuracy of patient critical care billing.

## Introduction

Emergency medicine (EM) physician compensation models often have a productivity component based on the relative value unit (RVU) [1-3]. RVUs demonstrate the value of a service or procedure relative to all services and procedures. The Current Procedural Terminology (CPT) codes assign RVU values. RVU dollar conversion factors determine the total reimbursement for a patient visit. From an educational perspective, it is essential that residents learn to utilize correct CPT codes for accurate billing and appropriate reimbursement.

Within EM residency training, formal education in billing and documentation varies greatly from program to program as there is no requirement by the Accreditation Council for Graduate Medical Education (ACGME) to provide such education. Several studies have shown that residents feel there is inadequate billing training within their formal curriculum [4-6]. A 2010 survey of EM residents revealed that 47% of residents reported inadequate billing and documentation training [4]. In a 2019 observational study of residents and staff physicians, the post-test survey revealed that 100% of residents and 79% of staff physicians desired more billing education during training [5]. Only 37% of residents felt confident in billing, and 94.9% felt that billing should be a part of training in a 2018 questionnaire [6]. In 2018, Bang and Bahl performed a randomized, prospective controlled study where a cohort of postgraduate year (PGY)-1 EM residents were given significant billing and coding education and feedback [7]. They reported that the intervention cohort billed a level 5 chart (CPT code 99285) 27% more often than the control cohort, with a level 5 chart being the highest value outside of critical care billing [7]. Similar improvements were seen in two recent studies focusing on surgical residents in the outpatient setting [8,9]. These data suggest that early billing education during residency training is both desired and improves billing knowledge and billing accuracy.

Considering the lack of billing education perceived by residents completing their training and the fact that physician compensation is intimately tied to sound documentation and billing practices, further research is needed to evaluate the effect of billing and documentation education during residency. In a broader sense, hospital revenue relies on accurate documentation and billing by physicians, and patients have a personal stake in accurate billing in cases of incorrect charting and overbilling. We hypothesized that after formal resident education on billing, residents would demonstrate improved billing performance.

For the purposes of this study, we focused on critical care time rather than total RVUs. We did this for several reasons. First, the scarcity of literature on critical care billing amounts to less knowledge about specific critical care billing by EM residents as compared to generalized RVU education. Second, generalized tracking of resident RVUs may be subject to additional confounding billing variables, making tracking of effective RVU billing educational initiatives more challenging. Third, we wanted to test an approach that would be relatively easy to replicate and track in other residency curriculums.

Critical care billing is justified (using CPT codes 99291 and 99292) when the criteria for critical care and critical care services are met [10-12]. The Centers for Medicare and Medicaid Services (CMS) and CPT both agree a patient must receive “direct delivery by a physician(s) medical care” such that the illness or injury must impair “one or more vital organ systems such that there is a high probability of imminent or life-threatening deterioration in the patient’s condition” to qualify as critical care [11]. These codes are both time and performance based. In fact, the following two criteria must be met. First, the patient’s condition must meet the definition of a critical illness or injury as described above. Second, the total critical care time delivered must be documented and must be a minimum of 30 minutes, exclusive to separately reportable procedure time [11].

## Materials and methods

Study setting and population

This study was conducted at an academic medical center’s single emergency department (ED) with adult and pediatric emergency facilities, along with a comprehensive stroke center, cardiac care center, and level 1 trauma center designations. The annual volume for the ED is approximately 90,000 patients. This department is home to a three-year EM Doctor of Medicine (MD)/Doctor of Osteopathic Medicine (DO) residency program with 14 residents per class.

Study design

This was a prospective observational study of an educational intervention for a cohort of EM residents. The purpose of this study was to determine whether introducing billing education during residency training would improve billing knowledge and accuracy. During the one-year intervention phase, two billing and documentation lectures were added to the residency didactic curriculum, and critical care billing was discussed in the semi-annual evaluation of each resident. In addition to lectures and discussions, monthly billing reports were sent to residents. To detect changes in critical care documentation on charts signed by all EM residents before and after the educational intervention, baseline data were collected from the third-party billing company for one academic year prior to (July 2019 to June 2020) and the academic year of (July 2020 to June 2021) the intervention. The protocol was reviewed and approved by the Institutional Review Board (IRB), package number 1753221.

An initial 25-minute billing lecture was given in July 2020 and reviewed general billing requirements with basics on documenting critical care billing time. This lecture was prepared by an EM faculty member with an interest in the topic. Resources used for preparing the didactic sessions included personal clinical experience, previous national coding and billing lectures attended by the faculty member, and utilization of online resources such as the documentation guidelines provided by the American College of Emergency Physicians (ACEP) reimbursement and coding committee. This lecture was aided by a PowerPoint (Microsoft Corporation, Redmond, WA) presentation and made available to all residents.

The second billing lecture in October 2020 provided an opportunity for spaced repetition of general billing rules. This lecture included an increased focus on critical care billing documentation with several case examples. Ultimately, the second lecture provided an opportunity to expose residents, particularly those excused from the July 2020 lecture to the topic. The second lecture, like the first, was presented with a PowerPoint presentation and sent to residents via email.

While residency didactics are required in our program, there are occasional excused absences for ACGME duty hour rules, illness, vacation, and other possibilities. Although attendance for these two billing lectures was not specifically tracked for this study, an email was sent to all residents following each billing lecture providing the PowerPoint lecture slides. Included in the email was a written, narrative summary of what was covered in each lecture to try and capture as many residents as possible to the billing education exposure.

To reinforce exposure, monthly feedback reports and discussion of performance during semi-annual evaluations were combined with a didactic intervention to optimize content delivery, encourage participation, and introduce a longitudinal element as demonstrated in other studies [7-9,13-16].

Study protocol

EM physician documentation is completed in the electronic medical record (EMR). At this ED, virtually all non-fast track notes are initiated and completed by residents, with attendings reviewing residents’ notes, making necessary amendments, and attesting. For this study, only EM resident notes completed on the adult side of the ED were included for analysis. Pediatric notes were excluded as the rate of both admissions and pediatric critical care time has historically been significantly lower in the pediatric population at our hospital. Non-EM resident notes, physician assistant notes, nurse practitioner notes, and any notes only signed by an attending EM physician without resident involvement were excluded from the analysis.

All resident notes signed and submitted to the third-party coding company R1 RCM, Incorporated (Murray, UT) were included. R1 provided data on critical care billing for resident notes prior to the attending physician amending and/or attesting to the final note to avoid attending physician supplementation or correction of notes, which would skew individual resident billing data. Thus, this study's data are based on preliminary notes signed only by the resident and not the formal charts attested to and signed by attending physicians for actual billing purposes.

The last element of our methodology, after protocol placement, was incorporating monthly feedback reports. Thus, all EM residents received monthly feedback reports via their university email. Monthly resident billing data in an Excel spreadsheet (Microsoft Corporation) were emailed out to all residents displaying the resident’s name, critical care volume successfully billed as compared to total patient volume, and erroneously billed critical care charts as determined by R1. Additionally, a monthly billing deficiency report of both regular and critical care charts was prepared by R1 and provided to the residents showing the resident name, medical record number of patients with deficient charts as determined by R1, and the reason for the deficiency leading to a “down-coded chart.” Common reasons for chart deficiencies provided by the billing company were "potential critical care missed" if the resident did not bill critical care time but the R1 felt the chart met criteria; not enough elements in the history of present illness or physical exam sections for non-critical care charts; and forgetting to populate past histories to include social and family histories for non-critical care charts. R1 also provided information on charts that "did not meet critical care criteria" for residents to review when, according to R1, the residents erroneously attempted to bill critical care time. By providing the medical record number, residents had the opportunity to review their deficient charts.

In April 2020, residents began receiving the unblinded, monthly feedback report of individual and aggregate residency critical care billing data from the previous month. Three months of lead time for the reports prior to the full intervention phase of semi-annual documentation discussions and formal billing didactic sessions ensured residents had time to digest their billing data and incorporate the reports into their documentation habits for future shifts. The billing report emailed to residents each month was an Excel spreadsheet. The spreadsheet contained each resident dataset viewable by all residents in a non-anonymous fashion and included calculated residency critical care averages listed next to individual resident averages. Although it is unusual to non-anonymize data for study group participants, extending this practice to a residency curriculum offered residents a chance to modify deficient behavior at a time when their skill sets are most malleable and are in line with real-life workplace encounters. Many non-academic EDs employ feedback systems for attending physicians where metrics are compared to colleagues to encourage improvement in areas of weakness.

In tandem with monthly feedback emails was the inclusion of formal critical care billing feedback during semi-annual evaluations. Data discussion with the residents by program leadership occurred during their semiannual reviews, along with the clinical feedback, goals, and milestones traditionally discussed at these meetings. These meetings provided an opportunity for residents to request clarification of the critical care billing process and documentation.

Measurement and analysis

Resident billing data during and after the educational intervention period were obtained from R1 for July 2020 through June 2021. Data analysis periods were chosen to mirror the academic year. Retrospective billing data from the academic year July 2019 to June 2020 established a pre-intervention baseline and billing data from the academic year July 2020 to June 2021 established the intervention and post-intervention period. All data for this study were extracted prior to attending physician modification and final attestation for billing purposes. Data collection included total ED patient volume, critical care volume, percent of critical care by total volume, cases billed as critical care that did not meet criteria as determined by R1, and percent of cases billed as critical care that did not meet criteria.

For this study’s analysis, resident data were anonymized by the residency coordinator serving as an honest broker, and labeled as only PGY-1, 2, and 3 levels (14 residents from each PGY level). The IRB recommended using an honest broker and not comparing individual resident data to focus on critical care billing trends across PGY levels and the residency as a whole both pre and post educational intervention. As part of our unique matriculate-in-graduate-out model, some residents graduated while others matriculated during this study. Our approach was to evaluate an improvement across the entire residency with a comparison of a PGY level of residency, rather than tracking an individual resident improvement.

Statistical methods

All statistical analyses were conducted at a significance level of 0.05 using SAS 9.4 (SAS Institute, Cary, NC). Paired t-tests were used to compare the billing performances of pre and post interventions. The first paired t-test assessed the significance of the change of successfully and erroneously billed critical care charts from the pre to post-intervention period for the entire resident group. The second paired t-test assessed the significance of the change of successfully and erroneously billed charts from the pre to post-intervention period by resident cohort year. One-way ANOVA analyzed differences between pre to post-intervention changes amongst the three resident cohorts.

## Results

The billing performances were measured by successfully billed and erroneously billed charts as determined by R1. Successful charts met the qualification for critical care billing as identified by R1, the third-party biller. Charts identified as erroneous indicate critical care billing was attempted by the resident, but the documentation was either inadequate or criteria were not met as determined by the third-party biller. Statistical analyses of their performance and the results are discussed below.

The results of the first paired t-test of the entire resident group charts are shown in Table 1. These indicate the pre-intervention (2019-2020) average of successfully meeting critical care billing criteria was 5.16%, with a standard deviation of 2.22%. In comparison, the post-intervention (2020-2021) average of successfully meeting critical care billing criteria was 10.66%, with a standard deviation of 4.51%. The overall percentage of critical care billing accuracy increased significantly after intervention with a t-value of -8.46 (pre to post) and p-value <0.0001. There were no statistically significant differences for erroneously billed charts in the pre and post comparison.

**Table 1:** Paired t-test on the percent of successful and erroneous critical care billing pre (2019-2020) to post (2020-2021) intervention for the resident group.

Variable	n	Mean	Std. dev.	Min	Max	t*-*value	P-value
Pre % successful	42	5.16%	2.22%	2.20%	10.05%	-8.46	<0.0001
Post % successful	42	10.66%	4.51%	4.51%	25.47%		
Pre % erroneous	42	1.43%	1.50	0.11	8.15	0.42	0.68
Post % erroneous	42	1.31%	1.04	0.00	4.75		

The results of the second paired t-test are indicated in Table 2. These data compare efforts by resident cohort year (PGY1, PGY2, and PGY3) rather than as a group. All three cohorts of residents showed increasing post-intervention success in meeting billing criteria in a statistically significant manner (PGY1, p-value = 0.0004; PGY2, p-value = 0.0002; and PGY3, p-value = 0.0004), as seen in Table 2. There were no statistically significant differences for erroneously billed critical care criteria in pre and post intervention for all three resident cohorts.

**Table 2:** Paired t-test on the percent of successful and erroneous critical care billing pre (2019-2020) to post (2020-2021) interventions for resident cohorts. PGY: postgraduate year.

Cohort	Variable	n	Mean	Std. dev.	Min	Max	t-value	p-value
PGY1	Pre % successful	14	3.16	0.58	2.2	4.37	-4.78	0.0004
	Post % successful	14	8.55	4.35	4.51	19.59		
PGY2	Pre % successful	14	5.65	2.22	2.57	9.7	-5.03	0.0002
	Post % successful	14	10.5	2.79	6.21	14.97		
PGY3	Pre % successful	14	6.68	1.81	4.34	10.05	-4.77	0.0004
	Post % successful	14	12.92	5.2	7.46	25.47		
PGY1	Pre % erroneous	14	0.75	0.41	0.11	1.65	-1.68	0.1161
	Post % erroneous	14	1.19	0.79	0.34	2.69		
PGY2	Pre % erroneous	14	1.53	1.15	0.31	3.92	1.02	0.3253
	Post % erroneous	14	1.12	0.91	0	2.87		
PGY3	Pre % erroneous	14	2.01	2.18	0.6	8.15	0.55	0.5894
	Post % erroneous	14	1.64	1.33	0.19	4.75		

A one-way ANOVA assessed the statistical significance of increased accurate billing amongst the cohorts. Table 3 shows the one-way ANOVA results comparing the billing accuracy of the different cohorts. There were no significant statistical differences between pre and post change for successfully and erroneously critical care billing in the three residence cohorts. Despite the reduction in error and increased accuracy in billing, these were not statistically significant.

**Table 3:** One-way ANOVA on pre (2019-2020) to post (2020-2021) intervention difference of percent for successful and erroneous critical care billing between resident cohort means. PGY: postgraduate year.

Pre to post difference	Code	n	Mean	Std. dev.	f-value	p*-*value
% successful	PGY1	14	-5.39	4.21	0.38	0.6862
	PGY2	14	-4.85	3.61		
	PGY3	14	-6.25	4.9		
% erroneous	PGY1	14	-0.44	0.97	1.01	0.3733
	PGY2	14	0.41	1.5		
	PGY3	14	0.37	2.5		

During the baseline year, from July 2019 through June 2020, residents saw 44,438 adult patients, as seen in Table 4, with 2,456 patients successfully billed for critical care. During the intervention period, from July 2020 through June 2021, residents saw 39,396 adult patients, with 4,305 patients successfully billed for critical care. The 5,042 reduction of patients seen residency-wide, compared to the baseline period, during the intervention period mostly impacted the PGY-3 cohort, which experienced a decrease of 4,719 patients alone.

**Table 4:** Raw values of pre and post-intervention periods, by group and cohorts, categorized by volume of patients, successful critical care billing, and erroneous critical care billing. PGY: postgraduate year.

	Patients	Successful	Erroneous
2019-2020			
Total	44,438	2456	666
PGY-1	10,729	338	79
PGY-2	14,376	822	216
PGY-3	19,333	1296	371
2020-2021			
Total	39,396	4305	529
PGY-1	10,989	955	132
PGY-2	13,793	1457	152
PGY-3	14,614	1893	245

## Discussion

As quality-of-care indices and productivity are increasingly tied to both hospital billing and physician compensation, education focused on coding and billing has become essential. This is particularly true given the increased complexity of billing and coding models, along with the now widespread implementation of the EMR. There are few studies assessing either the current state of billing education or models for improvement. Although the literature has recognized the importance of resident education in billing and documentation since at least 2005, several surveys demonstrated that resident physicians and recent graduates continue to feel unprepared [4-6,17].

Bang and Bahl performed a randomized controlled trial answering a similar question, but it was constrained by a small sample size of 11 residents, which further limited the total patient encounters to 1,181 [7]. While only an observational trial, our study supports Bang and Bahl’s prior findings of billing education effectiveness in the setting of more residents and more patient encounters [7]. Further, we focused on critical care documentation as opposed to total RVUs as another educational target to consider when incorporating billing education into residency curriculums. Finally, we utilized a unique matriculate-in-graduate-out model to track PGY level and residency-wide billing changes, rather than focusing on individual resident progression.

Our study demonstrated that despite a decrease in total overall ED volume by 5,042 (a loss of 11.3%) of patients during the intervention analysis period from July 2020 to June 2021, the rate of accurately billed critical care visits drastically increased post intervention by 1,849 patient charts (from 5.16% to 10.66%), as indicated in Table 4. In 2021, Medicare reimbursed $221.57 for CPT code 99291 (the first 30 to 74 minutes of critical care), and this would potentially translate to an additional $409,682.93 billed compared to the pre-intervention period depending on the hospital payor mix [18]. Additionally, despite a statistically significant increase in both the percentage and the absolute number of charts billed for critical care time, there was no increase in the number of inaccurately billed charts (666 charts or 1.43% pre-intervention vs. 529 charts or 1.31% post-intervention; p-value = 0.68). In fact, it is clear that all PGY levels, or resident cohorts, benefited similarly from the educational intervention, as shown in Table 3.

From a residency curriculum modification standpoint, our interventions were relatively simple to implement. Non-anonymous monthly feedback reports on resident critical care billing were requested from R1 and supplied to the residents via email to illustrate how they performed compared to their co-residents. Two 25-minute lectures on billing were added to the annual didactic calendar, along with planned discussion and feedback on critical care billing during semiannual evaluations between residents and program leadership. While it is difficult to know with certainty which of our interventions had a greater impact, we suspect it was the monthly feedback reports. The lectures provided residents with the building blocks they needed to bill successfully, but the feedback gave them a continuously updated sense of where they were in comparison to their peers and is not unlike other metric tracking distributed to EM physicians once they leave residency training. Although residents were not incentivized to improve their performance, we suspect a desire to perform near the residency mean led to the improvement of critical care billing.

While resident education was our primary study motivation, the positive effect on department revenue cannot be overlooked. Increasing effective billing per patient may slightly offset reduced revenue during similar events in the future [12,19]. Crafting a curriculum to optimize resident skillset marketability in an era of increasingly competitive employment practices may, in fact, serve as a boon to the native department.

As a final point worth mentioning, in 2023, significant national billing and documentation changes were made that placed more emphasis on the medical decision-making section of the note and less reliance on check-box tallying of a minimum number of elements from the history of present illness, physical exam, and review of systems sections to name a few. However, these billing changes did not affect the critical care documentation rules that this manuscript focuses on.

Limitations

Various limitations may have impacted the outcomes of the study. First, this study lacked a true control, and any sort of educational intervention performed within a single residency program cohort will be difficult to compare without potential conflation due to the sharing of new knowledge among participants in the study. Further, this study was designed as an observational study and we suspected, given the success of prior similar studies [4-8], that all residents could benefit from the educational material. We also assumed one class of residents would be largely similar to another class at an equal level of training. Next, there was no true isolated beginning or ending period cut off as residents had access to their performance three months before the defined intervention period to coincide with the academic year and semi-annual reviews. The three-month lead up allowed residents to view billing reports and digest that information as their status quo prior to the full intervention phase. In a future study, a multi-site strategy could be used to determine the effectiveness of documentation and billing interventions more fully.

One unanticipated limitation was the COVID-19 pandemic in the United States as it coincided, first, with the release of our monthly feedback reports and, second, with the intervention period [19]. The onset of the COVID-19 pandemic resulted in a substantial decrease in total ED volume not only at the location of our study but across the country, and EDs across the United States have yet to reach pre-pandemic levels [20]. It is difficult to say with certainty how this affected our study, without at the very least suggesting that the residents exposed to the early to mid-pandemic had vastly increased exposure to critical care early in their training. Despite this limitation, we must emphasize that while the absolute number and percentage of critical care cases billed increased, there was no decrease in critical care billing accuracy (1.43% pre-intervention vs. 1.31% post-intervention; p-value = 0.68 in Table 1).

## Conclusions

Residents often graduate feeling they did not receive enough formal education on documentation and billing. Given the interplay between physician documentation, billing, and compensation, residents require adequate training on this subject. Our analysis of critical care time billed by residents before and after the implementation of transparent critical care billing data for the residents, documentation lectures, and a feedback system from program leadership demonstrated that there was a statistically significant increase in critical care billing. This increased critical care billing was found despite a decrease in total ED volume during the intervention period. Additionally, critical care billing accuracy did not suffer, suggesting the study interventions led to appropriately increased critical care billing without a concomitant increase in erroneously submitted critical care codes. Future studies should consider multi-site projects where true controls can be used across several residency programs, as well as the evaluation of other aspects of documentation and billing. Our findings suggest that formal resident education in billing and documentation is easily integrated into current curriculums and that relatively straightforward interventions such as those in our study may measurably affect change in resident documentation and billing practices. As with all resident education initiatives, foundational knowledge in all aspects of healthcare to include documentation and billing will hopefully permeate the entire careers of resident physicians.
